# Release of exosomes in polytraumatized patients: The injury pattern is reflected by the surface epitopes

**DOI:** 10.3389/fimmu.2023.1107150

**Published:** 2023-03-09

**Authors:** Birte Weber, Dirk Henrich, Cora Rebecca Schindler, Ingo Marzi, Liudmila Leppik

**Affiliations:** Department of Trauma−, Hand− and Reconstructive Surgery, University Hospital Frankfurt, Goethe-University, Frankfurt, Germany

**Keywords:** polytrauma, extracellular vesicles, exosomes, size exclusion chromatography, thoracic trauma, traumatic brain injury, abdominal trauma, MACSPlex

## Abstract

**Background:**

Trauma is still a leading cause of morbidity and mortality, especially in the younger population. Trauma patients need a precise, early diagnostic to avoid complications like multiorgan failure and sepsis. Exosomes were described as markers and mediators in trauma. The aim of the present study was to analyze, whether the surface epitopes of plasma-exosomes can reflect the injury pattern in polytrauma.

**Material and Methods:**

Polytraumatized patients (Injury Severity Score = ISS ≥16, n = 38) were subdivided according to the predominant injury in either abdominal trauma, chest trauma or traumatic brain injury (TBI). Plasma exosomes were isolated via size exclusion chromatography. The concentration and size distribution of the plasma exosomes from emergency room samples were measured by nanoparticle tracking analysis. The exosomal surface antigens were investigated by bead-based multiplex flow cytometry and compared with healthy controls (n=10).

**Results:**

In contrast to other studies, we did not observe an increase in the total amount of plasma exosomes in polytrauma patients (1,15x109 vs. 1,13x109 particles/ml), but found changes in the exosomal surface epitopes. We found a significant reduction of CD42a+ (platelet-derived) exosomes in polytrauma patients, CD209+ (dendritic cell-derived) exosomes in the patients with predominant abdominal trauma, and CD11+ (monocyte-derived) exosomes in the patients with chest trauma. The group of patients with TBI was characterized in contrast by an increase of CD62p+ (endothelial/platelet-derived) exosomes (*p<0.05).

**Conclusion:**

Our data showed that the polytrauma injury pattern might be reflected by the cellular origin/surface epitopes of plasma-released exosomes immediately after trauma. The observed reduction of CD42+ exosomes in polytrauma patients was not associated with a reduction of total platelets in polytrauma patients.

## Introduction

Trauma is still a leading cause of morbidity and mortality worldwide (1). Polytraumatic injuries are complex and involve a combination of concomitant insults to multiple body regions and organs including large wounds and major tissue loss, often resulting in hemorrhage and a consecutive shock, prolonged systemic inflammation as well as major organ dysfunction and death. 6 % of polytrauma patients develop septic complications and up to 20% - multiorgan failure (2–4). These patients need a precise and quick diagnostic and an adequate therapy (5–7). The treatment of polytraumatized patients requires a large number of well-trained staff and expensive medical equipment and therefore results in a high economic burden for the society (8). One new promising tool for the diagnosis of trauma severity and complications could be the extracellular vesicles (EVs). EVs are defined as small particles released by cells, covered by a lipid-bilayer, but lack a functional nucleus (9). Historically, EVs were subdivided in 3 groups: the smallest population (size 30-150 nm) including the exosomes (or small EVs) formed by an endosomal route by healthy cells, the second group named microvesicles (MVs, size 100-800 nm), which are shed from the plasma membrane of healthy cells by budding, and the third group containing apoptotic bodies, which are released from non-vital cells by blebbing (size 200-5000 nm) (10–12). Modern research shows the heterogeneity of exosomes due to their diverse size, content, function, and biological origin, which complicates attempts to categorize them (reviewed in (13). Although no specific protein markers have been discovered to discriminate between the various EV types, MVs, exosomes, and apoptotic bodies have unique protein profiles as a result of their different biogenesis (14).

The increasing interest in extracellular vesicles is based on their ability to carry bioactive components and mediators for example RNA, miRNA, DNA or proteins and their important role in the cell-cell communication. It was shown that EVs surface antigens were reflecting their cellular origin and could help to understand the cellular communication mechanism after trauma (15). Recently, our working group reviewed the literature, which describes the roles of extracellular vesicles as mediators and markers of acute organ injury showed that our understanding of relationship between extracellular vesicles and polytrauma-mechanisms is still in its infancy (16, 17). One of the earliest observations in polytrauma research has been the observation that the amount of EVs released by platelets is significantly increased (18). The participation of platelets’ EVs in the modulation of vascular, leukocyte and platelet function were suggested in that study. The role of EVs as mediators of inflammation was further shown in an experimental chest trauma model (19). Recent studies characterized EVs in blood collected after traumatic injury and described their levels, cellular sources, adhesion molecule expression and procoagulant activity (18–21). In the present study we hypothesized that cellular origin/surface epitopes of plasma exosomes could reflect the polytrauma-injury pattern. In order to proof this hypothesis, we performed the broad surface epitopes characterization of exosomes from plasma of polytrauma patients and healthy controls using a multiplex bead-based platform. The obtained exosomes surface signature was then compared between subgroups of polytrauma patients with various injury patterns.

## Materials and methods

### Study design

All analyses were performed with ethical approval given by the Local Ethics Committee of the University of Frankfurt (approval ID 89/19). In the present study plasma-exosomes from multiple injured patients (ISS≥16, n=38) were compared with plasma exosomes from healthy volunteers (n=10, no medication, no inflammatory or other diagnosed diseases). The exosomes in the present study were defined by the presence of CD81, CD63 and CD9, as well as a size of ≤200nm. The study includes traumatized patients admitted from 2017-2020 to the Level 1 Trauma Center of the University Hospital of Frankfurt (Germany). Blood samples were taken at admission in the emergency room, immediately kept on ice and plasma was gained by 15 min centrifugation at 3500g and 4°C. The plasma samples from the healthy volunteers were further processed in the same way as patients’ samples. Two profiling analysis were performed. Initial analysis was performed with exosomes isolated from 10 polytrauma patients (ISS>16) and 10 healthy controls samples (**Supplementary Table S1**). In the second round of analysis another 28 polytrauma patients’ samples were enrolled. The patients were enrolled according to the major injury (Abbreviated Injury Scale = AIS ≥3, other body regions injury ≤2) in four groups (each n=7): polytrauma patients (ISS > 16; AIS ≥3 in more than 1 body region), polytrauma patients with TBI (ISS >16, AIS head ≥3), polytrauma patients with chest trauma (ISS > 16, AIS chest ≥3), polytrauma patients with abdominal trauma (ISS > 16, AIS abdomen AIS ≥3).

### Exosomes isolation and characterization

Exosomes were isolated from plasma by size exclusion chromatography (Exo-Spin TM, Exosome Size Exclusion Column, cell guidance systems, Cambridge, UK). Exosome particles’ number and size distribution of exosome particles were determined by nanoparticle tracking analysis (NTA) (Nanosight NS500, Malvern Panalytical, Kassel Germany). Protein concentration was measured by Coomassie Plus (Bradford) Asssay (Thermo Fisher Scientific, Rockford, IL, USA).

### Western blot

For Western blotting, exosome samples (2µg protein equivalent) were denatured in protein loading buffer (2xLaemmli buffer, Bio-Rad Laboratories, Hercules, CA, USA) at 95°C for 10 min. Proteins were separated by 10% sodium dodecyl polyacrylamide gel electrophoresis (SDS-PAGE), and were then transferred to polyvinylidene fluoride (PVDF) membranes (BioRad Laboratories, Hercules, CA, USA). The membranes were blocked with 5% non-fat milk in TBST buffer at +4°C overnight and afterwards incubated for 1 h at room temperature with antibodies against CD81 (Invitrogen, 10630D, 1:1000, Rockford, IL, USA), CD63 (Invitrogen, 10628D, 1:1000) and CD9 (Invitrogen, 10626D, 1:1000), followed by incubation with horseradish peroxidase-linked antibody (Cell signalling Technology, 7076, 1:2000, Leiden, The Netherlands) at room temperature for 1 h. Luminescent visualization was done using ECLTM Western-Blotting-Reagent (MERCK, RPN2016, Taufkirchen, Germany).

### Exosomes’ surface epitopes profiling

37 exosomal surface epitopes were quantified with the MACSPlex Exosome kit (Miltenyi Biotec, Bergisch Gladbach, Germany). The exosomes (20µg protein) from 10 samples of polytrauma patients, 10 samples of healthy control groups and 7 samples of each injury pattern subgroup were incubated with surface epitope-specific antibodies coupled with fluorescent labelled beads according to the manufactory instruction and analysed by flow cytometry analysis (FACS DIVA). For each group, the resulting APC-A values were normalized on Median APC-A values of total amount of EVs measured by CD63, CD81and CD9 and compared among the groups.

### Statistical analysis

All analysis were conducted with Graph Pad-Prism 9. The values are expressed as mean ± standard error of the mean (SEM). Normal distribution of the data was tested by Kolmogoroff-Smirnow-Lilleforts-Test. Data were analysed by one-way ANOVA followed by Dunnettes multiple comparison test. For statistical analysis of two groups, T-test was applied. Results were considered statistically significant when p ≤ 0.05.

## Results

### Polytrauma patients (n=10) vs. healthy volunteers (n=10)

**Figure 1:**
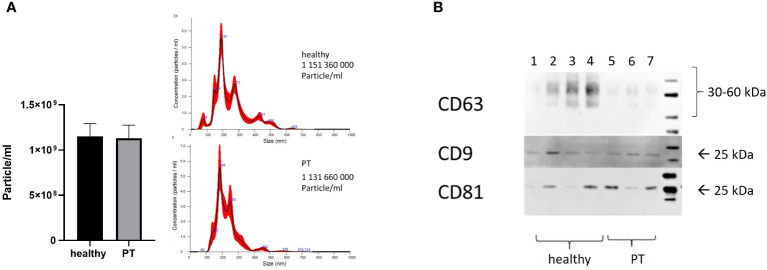
Characterization of exosomes. **(A)** Concentration and size distribution of exosomes isolated from patients and healthy volunteers’ plasma, nanoparticle tracking analysis (NTA); **(B)** CD63, CD81 and CD9 expression in plasma-isolated exosomes from healthy volunteers (Lines 1-4) and polytrauma patients (Lines 5-7), Representative Western-blot analysis.

The present study was conducted in 10 polytraumatized patients with an ISS of ≥16 and 10 healthy volunteers. The size and concentration of isolated exosomes were characterized by and NTA assay. The isolated exosomes showed the typical size of exosomes (≤200 nm, **Figures 1A, B**) and expressed exosome-specific tetraspanins CD9, CD8 and CD63 (**Figure 1B**). In contrast to the literature, we did not detect any significant difference in the exosome’s plasma-concentration (1,15x109 ± 4,5x108 vs. 1,13x109± 4,6x108 particles/ml) among healthy controls and polytraumatized patients (**Figure 1B**). The size distribution of the isolated exosomes was also similar in both investigated groups (**Figure 1B**).

**Figure 2:**
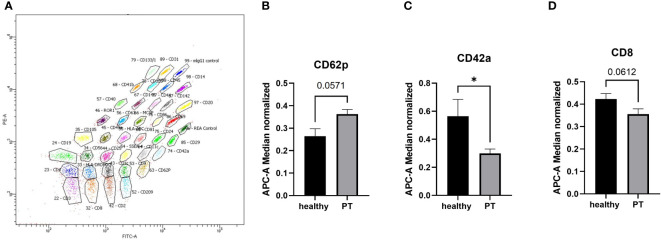
MACSPlex analysis of exosomal surface epitopes. **(A)** Representative image of MACSPlex exosomes flow cytometry analysis, polytrauma patient exosomes sample. **(B)** the higher amount of CD62p+ exosomes were detected in the plasma of polytrauma patients. **(C)** Amount of CD42a+ exosomes were significantly reduced in polytrauma patients as compared to the healthy controls. **(D)** the quantity of CD8+ exosomes were lower in patients’ plasma samples. PT = polytrauma (ISS≥16, n=10), healthy volunteers (n=10), *p <0.05. n = 10.

To analyze the surface epitopes of plasma exosomes and therefore the cellular origin of the exosomes, we conducted the bead‐based multiplex flow cytometry analysis for 37 different surface antigens (**Figure 2A**). Our results showed that out of 37 analyzed epitopes, CD42a was significantly (p<0.05) reduced in the polytrauma samples as compared to the healthy controls samples (**Figure 2C**). In the same time, CD62p was increased (**Figure 2B**) and CD8 was decreased (**Figure 2D**) in the polytrauma group, however not significant.

### Subgroup analysis of polytrauma patients with major injury (n=7, AIS≥3) vs. polytrauma patients (n=7, AIS ≥3 in more than 1 body region) vs. healthy volunteers (n= 10)

**Figure 3:**
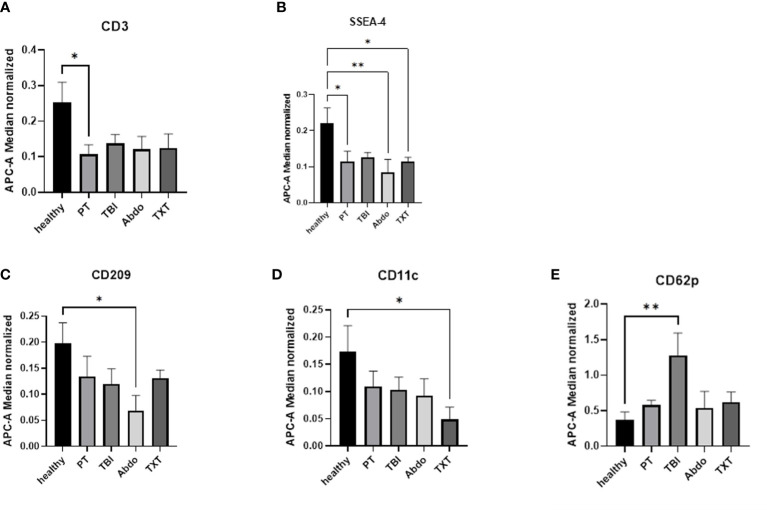
Exosomal surface epitopes expression in the patients’ subgroups. **(A)** The number of CD3+ exosomes were significantly reduced in polytrauma patients’ group. **(B)** Amount of SSEA-4+ exosomes were significantly reduced in all groups of patients except TBI. **(C)** CD209+exosomes are significantly reduced in abdominal trauma group of patients. **(D)** CD11c+ exosomes were significantly reduced in TXT patients’ group. **(E)** the number of CD62p+ exosomes were significantly increased in TBI patients. *p ≤ 0.05 and **p≤ 0.01. PT = polytrauma (n=7), healthy volunteers (n=10), TBI, traumatic brain injury (n=7); Abdo, abdominal trauma (n=7); TXT, thoracic trauma (n=7).

To verify if the expression of exosomal surface epitopes is different in patients with different injury patterns, we conducted MACSPlex data analysis in the subgroups of trauma patients. Patients were divided into 3 sub-groups: traumatic brain injury (TBI), abdominal trauma or thoracic trauma (TXT) by the type of leading trauma. The exosomes’ epitopes expression in these groups was compared with that in polytrauma patients with multiple injuries (no leading trauma focus, PT) and healthy controls. Out of 37 analyzed epitopes, 5 have shown differential expression in these patients’ sub-groups (**Figure 3**). As compared to the healthy controls, CD3 was found to be significantly reduced in the polytrauma group of patients (**Figure 3A**). SSEA-4 was reduced in all groups of patients, except the TBI group (**Figure 3B**). In the abdominal trauma group, CD209 was significantly reduced (**Figure 3C**), and in the chest trauma group of patients CD11c was significantly reduced (**Figure 3D**). In the TBI patients, the significant increase of CD62p+ exosomes were observed (**Figure 3E**).

## Discussion

Polytrauma patients still represent one of the most difficult group of patients in terms of diagnosis and treatment. For the most parts, understanding the complex pathophysiological steps after trauma are still unknown. Therefore, considerable efforts are directed to the search for new diagnostic parameters, which could reflect injury patterns and could help to provide straightforward treatment. Next to soluble mediators, such as cytokines commonly used as diagnostic parameters, extracellular vesicles found in circulating blood after severe trauma (15) could be an alternative. EVs are known to be released from cells into circulation blood as a reaction to the injury. At the same time, EVs are promoters of intercellular exchange due to their cargo and surface epitopes.

In the present study we hypothesized that exosomes, floating in the plasma of polytrauma patients and bearing the specific epitopes reflecting their cellular origin, could be a potential diagnostic parameter as they reflect the patients’ injury patterns. To investigate this hypothesis, we compared the exosomal surface epitopes in polytrauma patients and healthy controls.

In contrast to a previous experimental study, we did not find a difference in the total concentration of plasma exosomes (19) after trauma. This could be explained by differences in the patients’ cohorts studied and by discrepancies in the methods used to isolate EVs. In 2019, our working group demonstrated an increase in the total amount of EVs in trauma patients, but the study was performed on a specific subset of trauma patients: those with alcohol-induced intoxication and liver injury (22). Other studies described a significant increase of the total number of circulating EVs and/or changes in CD45+ EVs in patients after severe trauma when using different EVs isolation methods. These studies also most probably worked with different EV fractions (15, 18).

In our current analysis, we found a significant reduction of CD42a+ exosomes, which could be of megakaryocytes and platelets origin (**Figure 2**) in polytrauma patients. According to previous studies, platelet-derived EVs are a typical pattern in polytrauma patients (23, 24). However, the predictive potential of these specific EVs for trauma patients’ outcome is not well understood. The role of these EVs in polytrauma was investigated in haemorrhagic shock models in vivo. Platelets’ EV were found to decrease endothelial cell permeability and to restore endothelial cell junctions in a tail snip haemorrhage model (25). Furthermore, in a rat model of traumatic haemorrhagic shock these EVs were found to maintain hemodynamic stability and attenuate uncontrolled bleeding (26), making them a potential treatment tool to prevent severe trauma outcome. The observed reduction of CD42+ exosomes in polytrauma patients was not associated with a reduction of total platelets in polytrauma patients

Results of our study showed indeed that different subgroup of polytrauma patients had specific exosomal epitopes patterns, which supports our initial hypothesis. In the subgroup analysis, CD3+exosomes were significantly reduced in the general multiple injured group compared to healthy patients. This decrease might be a result of a T-lymphocyte depletion caused by T-cells- apoptosis (27) described after severe trauma (28).

We also found that the decrease in monocyte/macrophage-derived (CD11c+) exosomes could be indicative for a chest trauma in polytraumatized patients. In our results, CD11c+ exosomes counts were significantly lower in patients with chest trauma, while in the other trauma groups the number of these exosomes were the same as in healthy controls (**Figure 3**). A correlation between monocyte-derived EVs count and clinical outcome of trauma patients was established by Matsumoto et al. (21). They showed that in the acute phase in trauma patients an increased number of monocyte-derived EVs correlate with the ISS and APACHEII patients score. This suggest their importance in the pathogenesis of early SIRS following trauma (21). Previous studies have described the release of monocyte-and macrophage-derived EVs in the context of the development of ARDS through the upregulation of pro-inflammatory mediators via activation of NF-ĸB (29). In the context of chest trauma, Shi et al. (30) described that M1 macrophages but not M0-derived EVs induce macrophage polarization (30). Detailed studies are needed to further proof that monocyte/macrophage-derived exosomes indicate chest trauma and if they can potentially predict patient’s outcome.

Our findings suggest that increased levels of CD62p+ exosomes could more likely be a specific marker of TBI in severely injured patients rather than a general marker of polytrauma, as it was described in literature. In 2001, Ogura et al. showed that the amount of CD62p+ microparticles was significantly increased in the serum of severely injured patients as compared to the healthy volunteers (18). In our polytraumatized patients, the CD62p+ exosomes count was also higher as in control group but this difference is not statistically significantly. Interestingly, this count was significantly higher in the TBI subgroup of the polytrauma patients (**Figure 3E**). Little is known about the functions of CD62p+ exosomes in severe injury. These exosomes were shown to be released from endothelial cells, but also from platelets and megakaryocytes (31). Experimental studies in vivo and in vitro have shown that the release of particles from endothelial cells can be caused by chest trauma or as a result of endothelial cell incubation with a polytrauma cytokine cocktail (19). Future research should re-examine the relationship of these particles and different injury patterns and explore the role of these particles in TBI.

In the subgroup of patients with abdominal injury we also found specific exosomal epitopes pattern. This group of patients was characterized by a significant reduction of CD209+ EVs, which was not yet described in the literature. The role of dendritic cells-derived EVs in immune regulation and T-cell response has been shown in experimental studies (32), however, the functions of these particles in the mechanism of the body’s response to injury need to be explored.

Overall, the results of this explorative study in polytrauma patients showed that a major injury in polytrauma patients seem to be reflected in the plasma exosomes’ epitopes/cellular origin profiles. This supports the need for further more specific investigations of various exosomes from different organs or tissues. Studies should also cover patients with sepsis or hemorrhagic shock and other critical conditions. Future analysis could find new exosomal surface epitopes, which might help to understand the trauma pathophysiology or might correlate with the patient’s outcome, e.g. the development of sepsis.

## Conclusion

Our data reveal that polytrauma patients (ISS >16, regardless to the injury pattern) exosomes population is characterized by a reduction of CD42+ exosomes. Moreover, the subgroup analysis showed that the patients’ injury pattern might be reflected in the exosome population: abdominal trauma is reflected by a significant decrease of CD209+ exosomes, thoracic trauma by a significant decrease of CD11c+ exosomes and traumatic brain injury by a significant increase of CD62p+ exosomes. Future studies could identify new populations of exosomes by means of new epitopes, which might help to understand the trauma pathophysiology or might correlate with the patient’s outcome, e.g. the development of sepsis.

## Data availability statement

The original contributions presented in the study are included in the article/**Supplementary Material**. Further inquiries can be directed to the corresponding author.

## Ethics statement

The studies involving human participants were reviewed and approved by Ethics Committee of the University of Frankfurt (approval ID 89/19). The patients/participants provided their written informed consent to participate in this study.

## Author contributions

BW, DH, CS, LL and IM substantial contributed to the conception or design of the work. BW, LL, DH, CS are responsible for the analysis and the interpretation of data for the work. BW, DH, LL, IM drafting the work or revising it critically for important intellectual content. All authors provide approval for publication of the content and agree to be accountable for all aspects of the work in ensuring that questions related to the accuracy or integrity of any part of the work are appropriately investigated and resolved. All authors contributed to the article and approved the submitted version.
