# Supplementary material for: Release of exosomes in polytraumatized patients: The injury pattern is reflected by the surface epitopes
Source: Front Immunol. 2023 Mar 9;14:1107150. doi: 10.3389/fimmu.2023.1107150 (PMC10034046; doi:10.3389/fimmu.2023.1107150)
Supplement: Supplementary file 1 [file Table_1.docx]

| ; | Age (years) | Male/female (%) | ISS | HB (g/dl) | Lactate (mg/dl) | Death (%) | Time on ICU, IMC (days) | Ventilation (days) | Catecholamines (%) | Platelets (/nl) |
| --- | --- | --- | --- | --- | --- | --- | --- | --- | --- | --- |
| Polytrauma  n=10; ISS ≥16 | 41.7 (±15.9) | 80/20 | 31 (±11.9) | 11.6 (±2.7) | 25 (±13.7) | 30 | 11.4 (±9.0) | 5 (±5.7) | 70 | 186.5 (±85.4) |
| Healthy  n=10 | 38.6  (±9.0) | 30/70 | - | 13.5-17.5^#^ | 4.5-14.5^#^ | - | - | - | - | 146-328^#^ |

|  | Age (years) | Male/female  (%) | ISS | HB (g/dl) | Lactate (mg/dl) | Death (%) | Time on ICU, IMC (days) | Ventilation (days) | Catecholamines (%) | Platelets (/nl) |
| --- | --- | --- | --- | --- | --- | --- | --- | --- | --- | --- |
| Polytrauma  n=7 (2 regions AIS ≥3) | 30.6 (±11.8) | 85,7/14.3 | 31.7 (±7.2) | 9.7 (±1.9) | 22.7 (±12,0) | 14.3 | 21.3 (±18.4) | 15.3 (±20) | 85.7 | 217.7 (±190.7) |
| TBI  n=7; AIS ≥3 | 60 (±19.7) * | 28.6/71.4 | 22.3 (±8.8) | 11.8 (±2.2) | 26.2 (±8.5) | 14.3 | 11.7 (±6.2) | 3.5 (±5.5) | 0 | 246.9 (±38.2) |
| Abdo  n=7; AIS ≥3 | 52.8 (±17.2) | 83,3/16,7 | 20 (±4,7) * | 11.7 (±2.1) | 25.5 (12.1) | 0 | 13.5 (±18.4) | 6.0 (±9.5) | 66.6 | 283.8 (±119.9) |
| TXT  n=7; AIS ≥3 | 45.3 (±19.7) | 100/0 | 25.3 (±8.8) | 14.2 (±1.1) * | 17.9 (±5.3) | 0 | 10.3 (±5.5) | 3.3 (±3.3) | 62.5 | 228 (±52.4) |

PT= polytrauma; ICU = Intensive Care Unit, IMC = Intermediate Care. ^# -^ reference values; * = p<0.05 compared to PT
